# *N*′-(2-Chloro-5-nitro­benzyl­idene)-3-hydroxy­benzohydrazide methanol solvate

**DOI:** 10.1107/S1600536808019636

**Published:** 2008-07-05

**Authors:** Zhi Zhou

**Affiliations:** a Department of Chemistry, Kaili College, Kaili Guizhou 556000, People’s Republic of China

## Abstract

In the title compound, C_14_H_10_ClN_3_O_4_·CH_3_OH, the dihedral angle between the two benzene rings is 33.9 (2)°. In the crystal structure, the methanol solvent mol­ecules are linked to the Schiff base mol­ecules through inter­molecular N—H⋯O and O—H⋯O hydrogen bonds. Mol­ecules are further linked through inter­molecular O—H⋯O and O—H⋯N hydrogen bonds, forming chains running along the *b* axis.

## Related literature

For related structures, see: Zhou & Tang (2007 ▶); Zhou & Xiao (2007 ▶). For related literature, see: Ali *et al.* (2007 ▶); Butcher *et al.* (2007 ▶); He (2008 ▶); Jing & Yu (2007 ▶); Nie (2008 ▶).
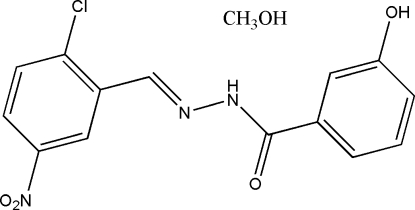


## Experimental

### 

#### Crystal data

C_14_H_10_ClN_3_O_4_·CH_4_O
*M* *_r_* = 351.74
Monoclinic, 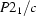

*a* = 7.716 (3) Å
*b* = 11.945 (2) Å
*c* = 17.650 (3) Å
β = 99.886 (2)°
*V* = 1602.6 (7) Å^3^
*Z* = 4
Mo *K*α radiation
μ = 0.27 mm^−1^
*T* = 298 (2) K
0.27 × 0.23 × 0.22 mm

#### Data collection

Bruker SMART CCD area-detector diffractometer
Absorption correction: multi-scan (*SADABS*; Bruker, 2001 ▶) *T*_min_ = 0.931, *T*_max_ = 0.943
12656 measured reflections
3316 independent reflections
2396 reflections with *I* > 2σ(*I*)
*R*_int_ = 0.034

#### Refinement

*R*[*F* ^2^ > 2σ(*F* ^2^)] = 0.045
*wR*(*F* ^2^) = 0.121
*S* = 1.01
3316 reflections
223 parameters
H atoms treated by a mixture of independent and constrained refinement
Δρ_max_ = 0.18 e Å^−3^
Δρ_min_ = −0.22 e Å^−3^

### 

Data collection: *SMART* (Bruker, 2007 ▶); cell refinement: *SAINT* (Bruker, 2007 ▶); data reduction: *SAINT*; program(s) used to solve structure: *SHELXTL* (Sheldrick, 2008 ▶); program(s) used to refine structure: *SHELXTL*; molecular graphics: *SHELXTL*; software used to prepare material for publication: *SHELXTL*.

## Supplementary Material

Crystal structure: contains datablocks global, I. DOI: 10.1107/S1600536808019636/rz2226sup1.cif

Structure factors: contains datablocks I. DOI: 10.1107/S1600536808019636/rz2226Isup2.hkl

Additional supplementary materials:  crystallographic information; 3D view; checkCIF report

## Figures and Tables

**Table 1:** Hydrogen-bond geometry (Å, °)

*D*—H⋯*A*	*D*—H	H⋯*A*	*D*⋯*A*	*D*—H⋯*A*
O4—H4⋯O3^i^	0.82	1.91	2.7237 (19)	169
O4—H4⋯N2^i^	0.82	2.62	3.118 (2)	120
O5—H5⋯O3^i^	0.82	2.00	2.817 (2)	175
N3—H3*B*⋯O5	0.81 (3)	2.05 (3)	2.854 (2)	173 (3)

Symmetry code: (i) 
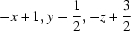
.

**Fig. 1.**
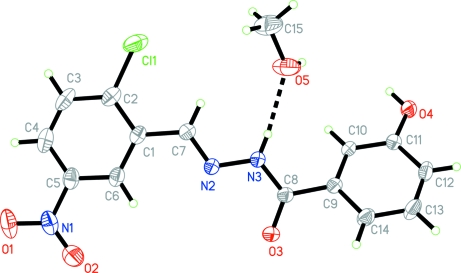
The molecular structure of the title compound, with displacement ellipsoids drawn at the 30% probability level. Hydrogen bond is shown as a dashed line.

**Fig. 2.**
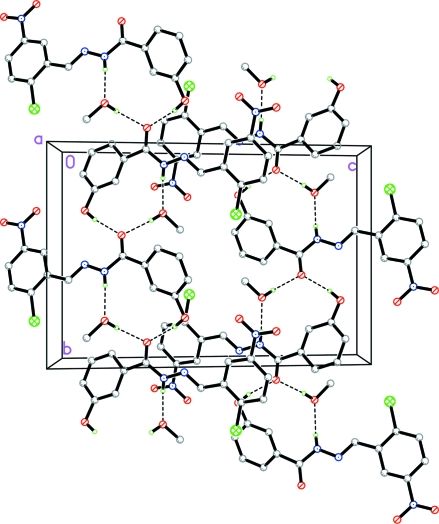
The crystal packing of the title copound, viewed along the a axis. Hydrogen bonds are shown as dashed lines.

## supplementary crystallographic information

### Comment

Recently, we have reported two metal complexes with Schiff base ligands (Zhou & Tang, 2007; Zhou & Xiao, 2007). We report herein the crystal structure of the title Schiff base compound (Fig. 1).

The title compound consists of a Schiff base molecule and a methanol molecule of crystallization. The dihedral angle between the two benzene rings is 33.9 (2)°. All bond lengths are comparable to those found in similar compounds (Ali *et al.*, 2007; Nie, 2008; He, 2008; Butcher *et al.*, 2007; Jing & Yu, 2007). In the crystal structure, the methanol molecules are linked to the Schiff base molecules through intermolecular N–H···O and O–H···O hydrogen bonds (Table 1). Molecules are further linked through intermolecular O—H···O and O—H···N hydrogen bonds to form chains running along the *b* axis (Fig. 2).

### Experimental

2-Chloro-5-nitrobenzaldehyde (1.0 mmol, 167.1 mg) and 3-hydroxybenzohydrazide (1.0 mmol, 152.1 mg) were dissolved in methanol (30 ml). The mixture was stirred at reflux for 30 min to give a colourless solution. After keeping the solution in air for a few days, colourless block-shaped crystals were formed.

### Refinement

H3B was located in a difference Fourier map and refined isotropically, with *U*_iso_(H) fixed at 0.08 Å^2^. Other H atoms were positioned geometrically and refined using a riding model with O–H = 0.92 Å, C–H = 0.93-0.96 Å, and with *U*_iso_(H) = 1.2 or 1.5*U*_eq_(C, O).

### Crystal data

**Table:**

C_14_H_10_ClN_3_O_4_·CH_4_O	*F*_000_ = 728
*M**_r_* = 351.74	*D*_x_ = 1.458 Mg m^−^^3^
Monoclinic, *P*2_1_/*c*	Mo *K*α radiation λ = 0.71073 Å
Hall symbol: -P 2ybc	Cell parameters from 2947 reflections
*a* = 7.716 (3) Å	θ = 2.3–24.5º
*b* = 11.945 (2) Å	µ = 0.27 mm^−^^1^
*c* = 17.650 (3) Å	*T* = 298 (2) K
β = 99.886 (2)º	Block, colourless
*V* = 1602.6 (7) Å^3^	0.27 × 0.23 × 0.22 mm
*Z* = 4	

### Data collection

**Table:**

Bruker SMART CCD area-detector diffractometer	3316 independent reflections
Radiation source: fine-focus sealed tube	2396 reflections with *I* > 2σ(*I*)
Monochromator: graphite	*R*_int_ = 0.034
*T* = 298(2) K	θ_max_ = 26.5º
ω scans	θ_min_ = 2.1º
Absorption correction: multi-scan(SADABS; Bruker, 2001)	*h* = −9→9
*T*_min_ = 0.931, *T*_max_ = 0.943	*k* = −14→14
12656 measured reflections	*l* = −21→22

### Refinement

**Table:**

Refinement on *F*^2^	Secondary atom site location: difference Fourier map
Least-squares matrix: full	Hydrogen site location: inferred from neighbouring sites
*R*[*F*^2^ > 2σ(*F*^2^)] = 0.045	H atoms treated by a mixture of independent and constrained refinement
*wR*(*F*^2^) = 0.121	*w* = 1/[σ^2^(*F*_o_^2^) + (0.0596*P*)^2^ + 0.2859*P*] where *P* = (*F*_o_^2^ + 2*F*_c_^2^)/3
*S* = 1.01	(Δ/σ)_max_ < 0.001
3316 reflections	Δρ_max_ = 0.18 e Å^−^^3^
223 parameters	Δρ_min_ = −0.22 e Å^−^^3^
Primary atom site location: structure-invariant direct methods	Extinction correction: none

### Special details

**Table:**

Geometry. All e.s.d.'s (except the e.s.d. in the dihedral angle between two l.s. planes) are estimated using the full covariance matrix. The cell e.s.d.'s are taken into account individually in the estimation of e.s.d.'s in distances, angles and torsion angles; correlations between e.s.d.'s in cell parameters are only used when they are defined by crystal symmetry. An approximate (isotropic) treatment of cell e.s.d.'s is used for estimating e.s.d.'s involving l.s. planes.
Refinement. Refinement of *F*^2^ against ALL reflections. The weighted *R*-factor *wR* and goodness of fit *S* are based on *F*^2^, conventional *R*-factors *R* are based on *F*, with *F* set to zero for negative *F*^2^. The threshold expression of *F*^2^ > σ(*F*^2^) is used only for calculating *R*-factors(gt) *etc*. and is not relevant to the choice of reflections for refinement. *R*-factors based on *F*^2^ are statistically about twice as large as those based on *F*, and *R*- factors based on ALL data will be even larger.

### Fractional atomic coordinates and isotropic or equivalent isotropic displacement parameters (Å^2^)

**Table:**

	*x*	*y*	*z*	*U*_iso_*/*U*_eq_	
Cl1	0.27705 (9)	0.68944 (5)	0.42450 (4)	0.0774 (2)	
O1	−0.1188 (3)	1.16001 (18)	0.30340 (10)	0.0978 (7)	
O2	0.0033 (3)	1.21390 (16)	0.41618 (12)	0.0859 (6)	
O3	0.4707 (2)	1.09327 (11)	0.70885 (8)	0.0623 (4)	
O4	0.6995 (2)	0.71117 (11)	0.91382 (8)	0.0607 (4)	
H4	0.6479	0.6690	0.8808	0.091*	
O5	0.6192 (3)	0.69826 (13)	0.66034 (11)	0.0897 (6)	
H5	0.5907	0.6645	0.6968	0.135*	
N1	−0.0240 (3)	1.14400 (19)	0.36561 (12)	0.0652 (5)	
N2	0.3857 (2)	0.95587 (12)	0.58923 (8)	0.0428 (4)	
N3	0.4964 (2)	0.92397 (13)	0.65533 (9)	0.0424 (4)	
C1	0.2405 (2)	0.90699 (16)	0.46380 (10)	0.0439 (5)	
C2	0.1934 (3)	0.82357 (18)	0.40857 (12)	0.0534 (5)	
C3	0.0788 (3)	0.8448 (2)	0.34054 (13)	0.0684 (7)	
H3	0.0502	0.7880	0.3045	0.082*	
C4	0.0082 (3)	0.9485 (2)	0.32639 (12)	0.0663 (7)	
H4A	−0.0690	0.9633	0.2810	0.080*	
C5	0.0531 (3)	1.03136 (19)	0.38052 (11)	0.0533 (5)	
C6	0.1661 (3)	1.01296 (17)	0.44857 (10)	0.0468 (5)	
H6	0.1927	1.0705	0.4841	0.056*	
C7	0.3601 (3)	0.88397 (16)	0.53602 (11)	0.0460 (5)	
H7	0.4179	0.8154	0.5428	0.055*	
C8	0.5280 (2)	0.99647 (15)	0.71415 (10)	0.0429 (4)	
C9	0.6354 (2)	0.95567 (15)	0.78653 (10)	0.0409 (4)	
C10	0.6158 (2)	0.84831 (15)	0.81342 (10)	0.0414 (4)	
H10	0.5366	0.7992	0.7849	0.050*	
C11	0.7141 (3)	0.81385 (16)	0.88285 (11)	0.0451 (5)	
C12	0.8325 (3)	0.88781 (18)	0.92424 (11)	0.0536 (5)	
H12	0.9003	0.8652	0.9704	0.064*	
C13	0.8499 (3)	0.99430 (19)	0.89738 (12)	0.0591 (6)	
H13	0.9295	1.0432	0.9259	0.071*	
C14	0.7518 (3)	1.03021 (17)	0.82898 (11)	0.0511 (5)	
H14	0.7633	1.1029	0.8116	0.061*	
C15	0.6536 (4)	0.6218 (2)	0.60542 (17)	0.0899 (9)	
H15A	0.6769	0.6613	0.5609	0.135*	
H15B	0.5535	0.5739	0.5911	0.135*	
H15C	0.7542	0.5775	0.6263	0.135*	
H3B	0.538 (3)	0.862 (2)	0.6593 (14)	0.080*	

### Atomic displacement parameters (Å^2^)

**Table:**

	*U*^11^	*U*^22^	*U*^33^	*U*^12^	*U*^13^	*U*^23^
Cl1	0.0721 (4)	0.0661 (4)	0.0918 (5)	0.0006 (3)	0.0077 (3)	−0.0362 (3)
O1	0.1037 (15)	0.1191 (16)	0.0612 (11)	0.0065 (12)	−0.0124 (10)	0.0357 (11)
O2	0.0977 (14)	0.0643 (11)	0.0857 (13)	0.0020 (10)	−0.0123 (11)	0.0069 (10)
O3	0.0989 (12)	0.0348 (8)	0.0464 (8)	0.0086 (7)	−0.0068 (8)	−0.0028 (6)
O4	0.0881 (12)	0.0414 (8)	0.0441 (8)	−0.0011 (7)	−0.0130 (7)	0.0061 (6)
O5	0.1570 (19)	0.0489 (10)	0.0750 (12)	0.0195 (10)	0.0527 (12)	0.0041 (8)
N1	0.0607 (12)	0.0783 (14)	0.0543 (12)	−0.0043 (10)	0.0038 (10)	0.0231 (11)
N2	0.0519 (10)	0.0387 (8)	0.0355 (8)	−0.0009 (7)	0.0013 (7)	−0.0009 (7)
N3	0.0523 (10)	0.0363 (8)	0.0357 (8)	0.0010 (7)	−0.0005 (7)	−0.0004 (7)
C1	0.0437 (11)	0.0531 (12)	0.0356 (10)	−0.0079 (8)	0.0087 (8)	−0.0059 (8)
C2	0.0507 (12)	0.0624 (13)	0.0489 (12)	−0.0078 (10)	0.0132 (10)	−0.0157 (10)
C3	0.0644 (15)	0.0928 (19)	0.0463 (13)	−0.0128 (14)	0.0050 (11)	−0.0305 (13)
C4	0.0610 (14)	0.097 (2)	0.0378 (11)	−0.0051 (13)	0.0000 (10)	−0.0035 (12)
C5	0.0506 (12)	0.0699 (14)	0.0396 (11)	−0.0102 (10)	0.0080 (9)	0.0053 (10)
C6	0.0508 (11)	0.0549 (12)	0.0343 (10)	−0.0117 (9)	0.0061 (8)	−0.0001 (8)
C7	0.0541 (12)	0.0415 (10)	0.0413 (10)	−0.0005 (9)	0.0054 (9)	−0.0035 (8)
C8	0.0526 (11)	0.0343 (10)	0.0403 (10)	−0.0041 (8)	0.0041 (9)	−0.0002 (8)
C9	0.0476 (11)	0.0382 (10)	0.0359 (9)	−0.0012 (8)	0.0041 (8)	−0.0030 (7)
C10	0.0477 (11)	0.0377 (10)	0.0359 (10)	−0.0013 (8)	−0.0012 (8)	−0.0065 (8)
C11	0.0540 (12)	0.0415 (11)	0.0376 (10)	0.0039 (8)	0.0020 (9)	−0.0016 (8)
C12	0.0553 (13)	0.0621 (13)	0.0379 (10)	−0.0032 (10)	−0.0075 (9)	−0.0019 (9)
C13	0.0626 (14)	0.0634 (14)	0.0462 (12)	−0.0232 (11)	−0.0049 (10)	−0.0094 (10)
C14	0.0627 (13)	0.0434 (11)	0.0449 (11)	−0.0141 (9)	0.0026 (10)	−0.0020 (9)
C15	0.109 (2)	0.0755 (18)	0.095 (2)	0.0083 (16)	0.0456 (18)	−0.0195 (16)

### Geometric parameters (Å, °)

**Table:**

Cl1—C2	1.732 (2)	C4—C5	1.378 (3)
O1—N1	1.225 (2)	C4—H4A	0.9300
O2—N1	1.214 (3)	C5—C6	1.375 (3)
O3—C8	1.236 (2)	C6—H6	0.9300
O4—C11	1.355 (2)	C7—H7	0.9300
O4—H4	0.8200	C8—C9	1.481 (2)
O5—C15	1.390 (3)	C9—C10	1.385 (3)
O5—H5	0.8200	C9—C14	1.389 (3)
N1—C5	1.477 (3)	C10—C11	1.388 (3)
N2—C7	1.263 (2)	C10—H10	0.9300
N2—N3	1.376 (2)	C11—C12	1.385 (3)
N3—C8	1.342 (2)	C12—C13	1.372 (3)
N3—H3B	0.81 (3)	C12—H12	0.9300
C1—C6	1.397 (3)	C13—C14	1.379 (3)
C1—C2	1.398 (3)	C13—H13	0.9300
C1—C7	1.466 (3)	C14—H14	0.9300
C2—C3	1.387 (3)	C15—H15A	0.9600
C3—C4	1.358 (4)	C15—H15B	0.9600
C3—H3	0.9300	C15—H15C	0.9600
			
C11—O4—H4	109.5	C1—C7—H7	119.5
C15—O5—H5	109.5	O3—C8—N3	122.02 (17)
O2—N1—O1	123.6 (2)	O3—C8—C9	120.89 (16)
O2—N1—C5	118.83 (18)	N3—C8—C9	117.09 (16)
O1—N1—C5	117.5 (2)	C10—C9—C14	120.50 (17)
C7—N2—N3	115.93 (16)	C10—C9—C8	121.50 (16)
C8—N3—N2	118.75 (15)	C14—C9—C8	117.94 (17)
C8—N3—H3B	120.6 (18)	C9—C10—C11	120.11 (17)
N2—N3—H3B	120.6 (18)	C9—C10—H10	119.9
C6—C1—C2	117.57 (18)	C11—C10—H10	119.9
C6—C1—C7	120.92 (17)	O4—C11—C12	117.23 (17)
C2—C1—C7	121.49 (18)	O4—C11—C10	123.60 (17)
C3—C2—C1	121.6 (2)	C12—C11—C10	119.16 (18)
C3—C2—Cl1	118.32 (17)	C13—C12—C11	120.26 (18)
C1—C2—Cl1	120.09 (16)	C13—C12—H12	119.9
C4—C3—C2	120.1 (2)	C11—C12—H12	119.9
C4—C3—H3	119.9	C12—C13—C14	121.29 (18)
C2—C3—H3	119.9	C12—C13—H13	119.4
C3—C4—C5	118.8 (2)	C14—C13—H13	119.4
C3—C4—H4A	120.6	C13—C14—C9	118.66 (19)
C5—C4—H4A	120.6	C13—C14—H14	120.7
C6—C5—C4	122.5 (2)	C9—C14—H14	120.7
C6—C5—N1	118.48 (19)	O5—C15—H15A	109.5
C4—C5—N1	119.0 (2)	O5—C15—H15B	109.5
C5—C6—C1	119.39 (19)	H15A—C15—H15B	109.5
C5—C6—H6	120.3	O5—C15—H15C	109.5
C1—C6—H6	120.3	H15A—C15—H15C	109.5
N2—C7—C1	120.94 (18)	H15B—C15—H15C	109.5
N2—C7—H7	119.5		

### Hydrogen-bond geometry (Å, °)

**Table:**

*D*—H···*A*	*D*—H	H···*A*	*D*···*A*	*D*—H···*A*
O4—H4···O3^i^	0.82	1.91	2.7237 (19)	169
O4—H4···N2^i^	0.82	2.62	3.118 (2)	120
O5—H5···O3^i^	0.82	2.00	2.817 (2)	175
N3—H3B···O5	0.81 (3)	2.05 (3)	2.854 (2)	173 (3)

Symmetry codes: (i) −*x*+1, *y*−1/2, −*z*+3/2.
